# Case Report: Case report: Aslanger’s sign in electrocardiogram.

**DOI:** 10.12688/f1000research.156313.1

**Published:** 2024-10-15

**Authors:** Haifa Bradai, Sondes Laajimi, Rabeb Mbarek, Nabil Chebbi, Dorra Loghmari, Mounir Naija, Naoufel Chebili

**Affiliations:** 1 Department of Emergency Medical Services, Sahloul Hospital, Sousse, Tunisia

**Keywords:** Arterial pulse–tapping artifact, electrocardiographic artifact, pseudo-myocardial infarction.

## Abstract

Electrocardiograms (ECGs) can be affected by various factors and technical problems. It is rare for an artefact to be the cause of ST-segment elevation, especially in asymptomatic patients. An important distinction between true ST segment elevation caused by myocardial infarction and an artefact is that the baseline elevation in an artefact may begin before or after the appearance of the QRS complex. When confronted with an abnormal ECG with suspicious waveform contours and possibly only one completely normal limb lead, the diagnosis of arterial pulse artefact should be considered. It is important to exclude subjective assessments unless they are clearly labelled as such.

When encountering an abnormal ECG with suspicious waveform contours and possibly only one completely normal limb lead, the diagnosis of arterial pulse artefact should be considered.

## Introduction

It should be noted that artefacts on an electrocardiogram can result from a variety of causes, both internal and external. These include muscle tremors, the use of dry electrode gel and loose leads, and electromagnetic interference. These artifacts can sometimes mimic ECG abnormalities, which can cause problems for patient care.

In this report, we describe an unusual ECG artifact that caused large and bizarre T-waves on the ECG, The observed changes are aligned with those commonly associated with primary repolarisation changes characteristic of acute coronary syndrome. The artefact in question is caused by the overlapping of the artery pulse; this can be avoided by ensuring that the correct position is maintained. This is also known as the electromechanical association artefact.

## Case report

A 68-year-old man with no medical history and no cardiovascular risk factors consulted the emergency department of a district hospital due to 48 hours of atypical and paroxysmal chest pain (tingling). His physical examination revealed no abnormalities, with SBP at 120 mmHg, DBP at 50 mmHg, HR at 99 bpm, RR at 20/min, Sat O _2_ at 96% on air, T° at 37°C, and finger blood sugar at 0.9 g/l.

**Figure 1.**
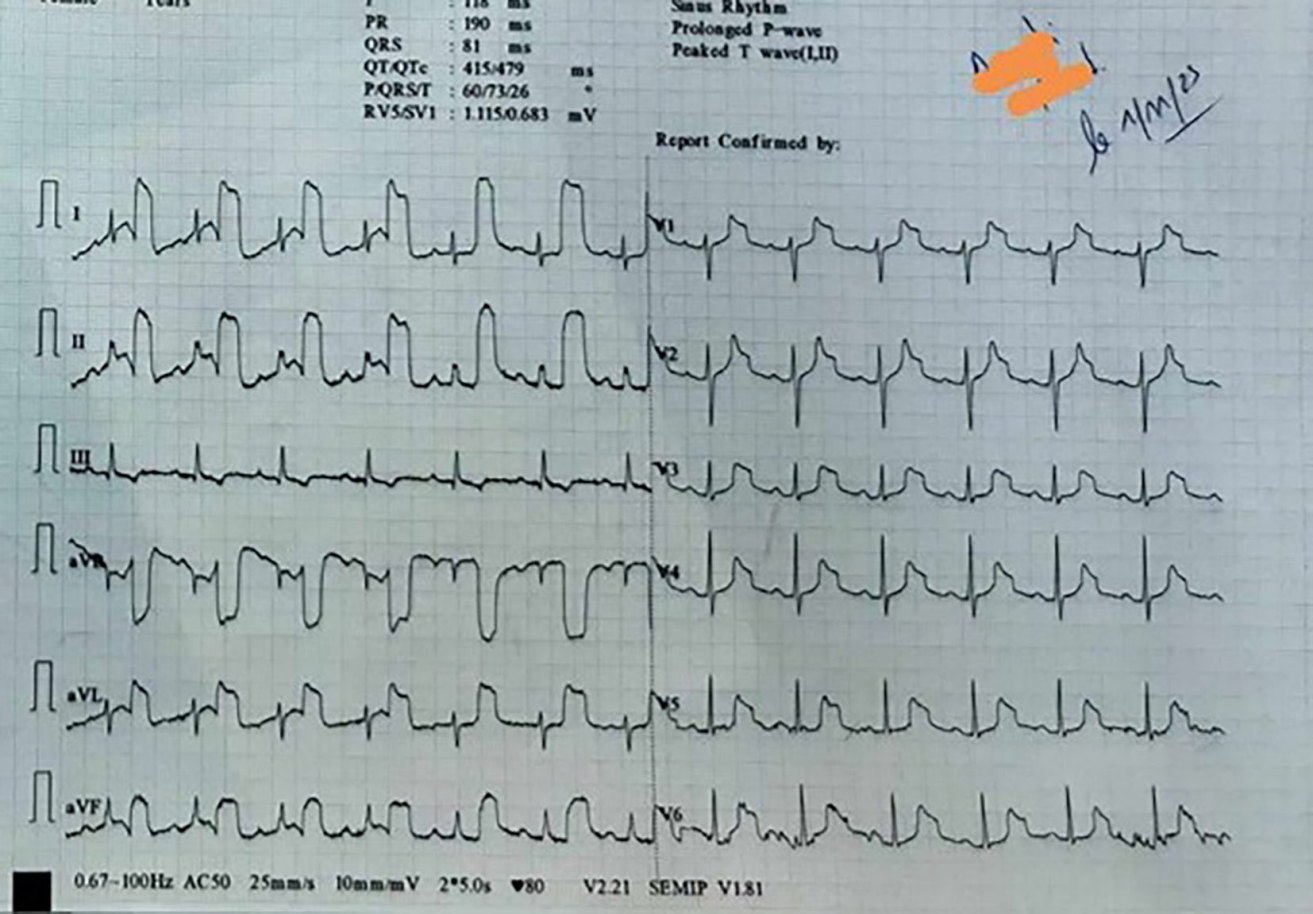
The prehospital 12-lead ECG.

Figure 1 shows the patient’s first ECG.

**Figure 2.**
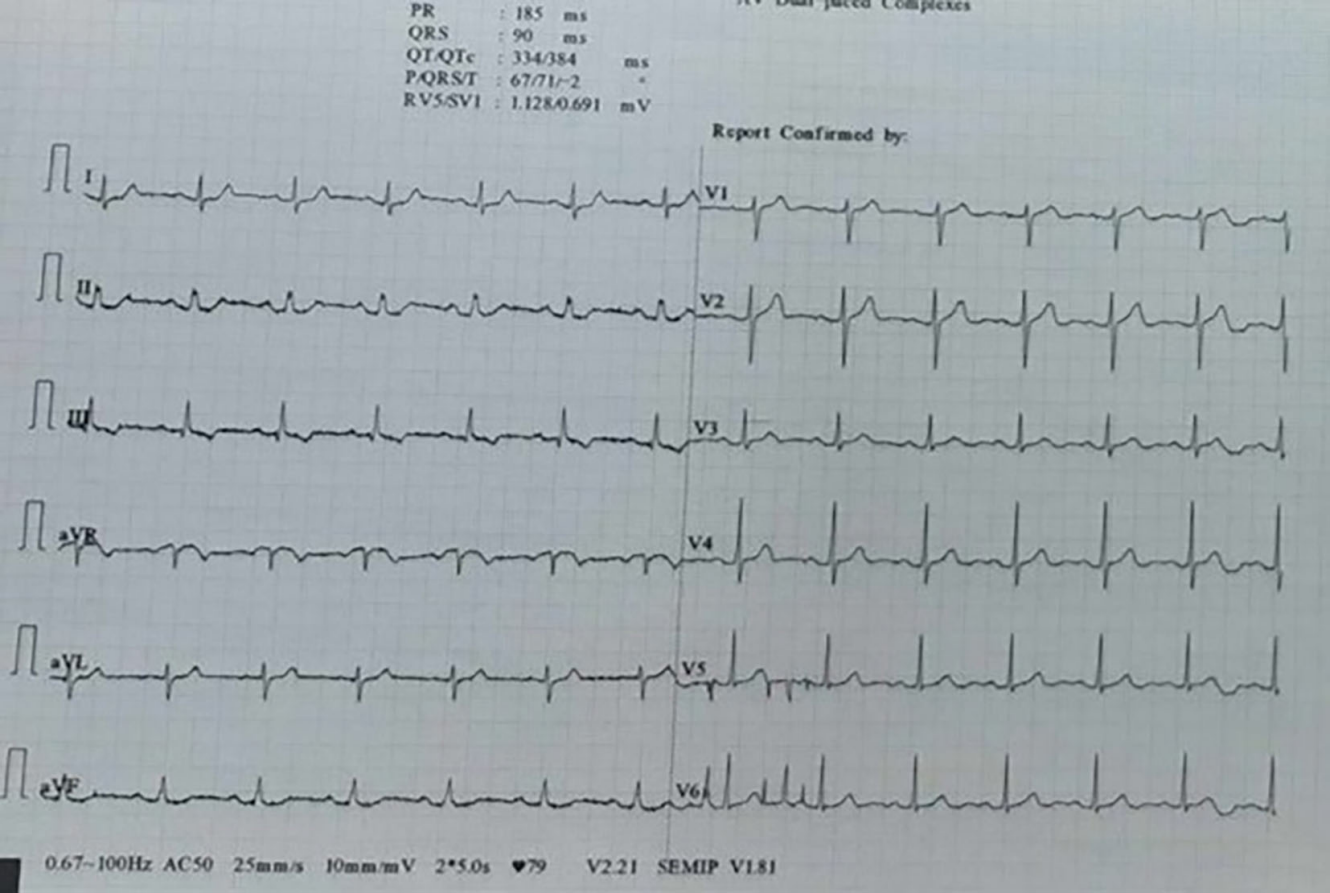
Normal ECG in ED.

The 12-lead ECG revealed a normal sinus rhythm with a heart rate of 80 beats per minute and normal axis. Abnormal T waves with bizarre morphology were observed in leads I, II, aVL, aVR, aVF, and from V1 to V6 in precordial leads. This abnormality was also observed in the 11-lead ECG, except for lead III, which was normal The emergency department physician’s interpretation of the T waves was that they were indicative of an ischaemic hyperacute condition. The patient was transferred to the emergency department (ED) at the university hospital due to suspected acute coronary syndrome. However, the ECG performed in the ED was completely normal ( Figure 2), and the high-sensitivity cardiac troponin (hs-cTn) levels were twice negative. Therefore, the pre-hospital ECGs were recognized as artifacts, and the patient was discharged home.

## Discussion

In this case, the electrocardiogram (ECG) showed anomalous T waves with unusual morphology in leads I, II, aVL, aVR, aVF, and V1 to V6. This distinct pattern was observed in the 11-lead ECG, with the exception of lead III. The presence of an arterial pulse-tapping artifact, also known as electromechanical association (EMA) or Aslanger’s sign, was indicated by the synchronous occurrence of this abnormality with the cardiac cycle. Aslanger initially described this phenomenon, ^1^ which some authors refer to as Aslanger’s sign. ^2^

The EMA artifact arises from the transmission of arterial pulsations, typically from the radial artery, onto the lead clips, generating aberrations in the ECG waveform. Contemporary electrocardiogram machines only record lead I and lead II, deriving the waveforms for other leads from these two. However, the majority of limb leads and augmented leads are susceptible to artifact. A consistent feature of EMA artifacts is the sparing of one lead, contingent on the limb generating the artifact. This serves as a crucial diagnostic clue, as outlined by Aslanger. ^3^ In this case, lead III was unaffected as it represents an ECG recording between the left arm and left leg. Therefore, we concluded that the source of the artifact was the right arm. When the clip was placed proximally during a repeat ECG in the emergency department, the 12-lead ECG exhibited no artifacts.

Aslanger’s sign has been recently described and there are limited reported cases in the literature. It is important to emphasise the potential risks associated with this condition, as it can mimic symptoms of acute coronary syndrome. This can lead to unnecessary invasive investigations if not promptly recognised. The artifact, induced by the mechanical tapping of the pulse on the ECG electrode, is in synchrony with the cardiac cycle and can manifest as ST segment changes (elevation or depression) accompanied by peculiar T waves. ^4, 5^

## Conclusion

This report presents a case of Aslanger sign, also known as arterial pulse-tapping ECG artifact. This anomaly presents as a primary repolarisation abnormality that is comparable to those observed in acute coronary syndrome.

It is important to be aware that an EMA artefact will almost always spare one of the limb leads, as this is a key factor in diagnosing it, which helps physicians avoid misinterpretation and unnecessary explorations.

## Declarations

### Participants’ consent

Informed written consent for participation in the study was obtained.

### Consent to publish

Written informed consent for publication of his clinical details and/or clinical images was obtained from the patient.

## Data Availability

No data are associated with this article.
