# Supplementary material for: Case Report: Case report: Aslanger’s sign in electrocardiogram
Source: F1000Res. 2024 Nov 27;13:1233. Originally published 2024 Oct 15. [Version 2] doi: 10.12688/f1000research.156313.2 (PMC11628937; doi:10.12688/f1000research.156313.2)
Supplement: Peer review history

# Reviewer report: Reviewer response for version 2

**DOI:** 10.5256/f1000research.174851.r343919

**Published:** 2024-12-09

**Authors:** Nihal Sheriff

**Affiliations:** 1 Madras Medical College, Chennai, India

No further comments to make.

Are enough details provided of any physical examination and diagnostic tests, treatment given and outcomes?

Partly

Is the case presented with sufficient detail to be useful for other practitioners?

Partly

Is sufficient discussion included of the importance of the findings and their relevance to future understanding of disease processes, diagnosis or treatment?

Partly

Is the background of the case’s history and progression described in sufficient detail?

Yes

Reviewer Expertise:

ECG

I confirm that I have read this submission and believe that I have an appropriate level of expertise to confirm that it is of an acceptable scientific standard.

# Reviewer report: Reviewer response for version 2

**DOI:** 10.5256/f1000research.174851.r343918

**Published:** 2024-12-05

**Authors:** Koji Takahashi

**Affiliations:** 1 Ehime University Graduate School of Medicine, Ehime, Japan

I have no comments.

Are enough details provided of any physical examination and diagnostic tests, treatment given and outcomes?

Yes

Is the case presented with sufficient detail to be useful for other practitioners?

Yes

Is sufficient discussion included of the importance of the findings and their relevance to future understanding of disease processes, diagnosis or treatment?

Partly

Is the background of the case’s history and progression described in sufficient detail?

Yes

Reviewer Expertise:

NA

I confirm that I have read this submission and believe that I have an appropriate level of expertise to confirm that it is of an acceptable scientific standard.

# Reviewer report: Reviewer response for version 1

**DOI:** 10.5256/f1000research.171603.r336519

**Published:** 2024-11-06

**Authors:** Koji Takahashi

**Affiliations:** 1 Ehime University Graduate School of Medicine, Ehime, Japan

I believe that Aslanger's sign is still not well recognized, even by medical staffs working in cardiovascular and emergency medicine. Therefore, it is worthwhile to case report this typical case.

 The abstract, introduction section, and case presentation section are good and concise.

 As described by the authors, Aslanger's sign has a strange waveform spanning ST to TP segments in 11 out of 12 ECG inductions. Nevertheless, there is an obvious reason for its occurrence, and if there is no word limit, it would be better to describe it.

Are enough details provided of any physical examination and diagnostic tests, treatment given and outcomes?

Yes

Is the case presented with sufficient detail to be useful for other practitioners?

Yes

Is sufficient discussion included of the importance of the findings and their relevance to future understanding of disease processes, diagnosis or treatment?

Partly

Is the background of the case’s history and progression described in sufficient detail?

Yes

Reviewer Expertise:

NA

I confirm that I have read this submission and believe that I have an appropriate level of expertise to confirm that it is of an acceptable scientific standard, however I have significant reservations, as outlined above.

## References

[1] : Mystery of “bizarre electrocardiogram” solved. *Journal of Electrocardiology* .2011;44(6) : 10.1016/j.jelectrocard.2011.04.001 810-811 10.1016/j.jelectrocard.2011.04.001 21658709

[2] : An unexpected ECG finding. *BMJ* .2023;380: 10.1136/bmj-2022-072333 e072333 10.1136/bmj-2022-072333 36863733

[3] : Unfamiliar waveforms spanning from the ST to TP segments only observed in certain limb leads of the standard 12-lead electrocardiogram due to Aslanger' s sign. *J Geriatr Cardiol* .2023;20(9) : 10.26599/1671-5411.2023.09.005 693-696 10.26599/1671-5411.2023.09.005 37840628 PMC10568546

# Response

**Published:** 2024-11-14

**Authors:** rabeb mbarek

**Affiliations:** emergency medical services, Sahloul Hospital, Sousse, Sousse, Tunisia

I would like to thank the reviewer for his precious time to read critically our case and for this important corrections suggested to improve the quality of our work.

 corrections about references done .

 Best regards

# Reviewer report: Reviewer response for version 1

**DOI:** 10.5256/f1000research.171603.r336521

**Published:** 2024-10-31

**Authors:** Nihal Sheriff

**Affiliations:** 1 Madras Medical College, Chennai, India

The authors here have compiled a case report of an acute coronary syndrome mimic – “Aslanger sign” which has quite recently received attention in the field of cardiology. They have done a good job in summarising the necessary points regarding the same. That said, the following parts may need a bit explaining/ corrections for the report to be more informative to the audience.

 In the **abstract** section, the sentence regarding suspicious waveform contours with one completely normal limb lead appears to be repeated. The fact to exclude subjective assessments can be better framed for easy understanding.

 The **introduction **section seems to be crisply constructed. Just one suggestion here would be to reframe the sentence as "the artefact can be avoided by moving the lead away from the pulsating artery".

 In the **case report** section, the history seems enough. However, the necessary investigations that need to be done in such a case seem to be lacking or have not been provided by the authors. Since the Aslanger sign is seen in hyperdynamic conditions, it would have been informative if the authors had disclosed the relevant blood investigations to rule out hyperthyroidism, cirrhosis or severe anemia (Takahashi K et al. 2023 [Ref - 1]) and also considering the fact that the patient had a pulse pressure of 70mmHg, it would have been prudent to rule out aortic regurgitation by echocardiography.

 A minor correction here would be “the abnormality was observed in all the leads except for lead III..” rather than "11-lead ECG" 

 The **discussion** part could be more detailed regarding the genesis of the sign with reference to the Einthoven triangle and probably an image depicting the same. Other corrections include “12-lead ECG” rather than “11-lead ECG” and the fact that the artefact arises not only from the radial artery but also from the posterior tibial artery (Takahashi K et al. 2023 [Ref - 2]).

 The **conclusion** seems to be well done.

 Overall, the case report brings about a novel and rare ECG finding that is of utmost importance to be recognised in the emergency department, although the corrections and suggestions, if included would make it more productive.

Are enough details provided of any physical examination and diagnostic tests, treatment given and outcomes?

Partly

Is the case presented with sufficient detail to be useful for other practitioners?

Partly

Is sufficient discussion included of the importance of the findings and their relevance to future understanding of disease processes, diagnosis or treatment?

Partly

Is the background of the case’s history and progression described in sufficient detail?

Yes

Reviewer Expertise:

ECG

I confirm that I have read this submission and believe that I have an appropriate level of expertise to confirm that it is of an acceptable scientific standard, however I have significant reservations, as outlined above.

## References

[1] : Unfamiliar waveforms spanning from the ST to TP segments only observed in certain limb leads of the standard 12-lead electrocardiogram due to Aslanger' s sign. *J Geriatr Cardiol* .2023;20(9) : 10.26599/1671-5411.2023.09.005 693-696 10.26599/1671-5411.2023.09.005 37840628 PMC10568546

[2] : Aslanger's sign in 12-lead electrocardiogram. *Oxf Med Case Reports* .2023;2023(3) : 10.1093/omcr/omad017 omad017 10.1093/omcr/omad017 36993830 PMC10041945

# Response

**Published:** 2024-11-14

**Authors:** rabeb mbarek

**Affiliations:** emergency medical services, Sahloul Hospital, Sousse, Sousse, Tunisia

I would like to thank the reviewer for his precious time to read critically our case and for his important corrections suggested to improve the quality of our work. We tried to respond to all of your suggestions.

 Here is a response to each part of your report

 In the **abstract** section, the sentence regarding suspicious waveform contours with one completely normal limb lead appears to be repeated. The fact to exclude subjective assessments can be better framed for easy understanding : the sentence repeated was deleted

 The **introduction **section seems to be crisply constructed. Just one suggestion here would be to reframe the sentence as "the artefact can be avoided by moving the lead away from the pulsating artery": reformulated sentence

 In the **case report** section, the history seems enough. However, the necessary investigations that need to be done in such a case seem to be lacking or have not been provided by the authors. Since the Aslanger sign is seen in hyperdynamic conditions, it would have been informative if the authors had disclosed the relevant blood investigations to rule out hyperthyroidism, cirrhosis or severe anemia (Takahashi K et al. 2023 [Ref - 1]) and also considering the fact that the patient had a pulse pressure of 70mmHg, it would have been prudent to rule out aortic regurgitation by echocardiography : in blood investigations no cirrhosis or severe anemia were observed. an investigation for hyperthyroidism was not done but no history of hyperthroidism for our patient. an echocardiography was done in the emergency room without abnormality ( This part was added in our case report).

 A minor correction here would be “the abnormality was observed in all the leads except for lead III..” rather than "11-lead ECG" : correction done

 The **discussion** part could be more detailed regarding the genesis of the sign with reference to the Einthoven triangle and probably an image depicting the same. Other corrections include “12-lead ECG” rather than “11-lead ECG” and the fact that the artefact arises not only from the radial artery but also from the posterior tibial artery (Takahashi K et al. 2023 [Ref - 2]) : corrections done

 Best regards
